# The efficacy of dynamic compression locking system vs. dynamic hip screw in the treatment of femoral neck fractures: a comparative study

**DOI:** 10.1186/s12891-022-05631-z

**Published:** 2022-07-12

**Authors:** Jian-Zhong Chang, Ya-Ping Xiao, Ling Li, Ming-Jian Bei

**Affiliations:** 1 Department of Orthopedic Surgery, CR & WISCO General Hospital, Wuhan University of Science and Technology, No. 209, Yejin Avenue, Hubei Province 430080 Wuhan, P.R. China; 2 Department of Orthopedic Surgery, Tongren Hospital of Wuhan University, Wuhan Third Hospital, No. 241, Pengliuyang Road, Hubei Province 430000 Wuhan, P.R. China; 3 Department of Traumatology, Beijing Ji Shui Tan Hospital, Xinjiekoudongjie 31, Xicheng dis, 100035 Beijing, P.R. China

**Keywords:** Femoral neck fractures, Internal fixation, Surgery, Clinical research

## Abstract

**Background:**

There is still a lack of consensus on which internal fixation method can better maintain the stability of femoral neck fractures (FNF), promote fracture healing, and reduce postoperative complications such as femoral head necrosis and nonunion. Therefore, the purpose of this study was to evaluate the clinical efficacy of the novel dynamic compression locking system (DCLS) versus dynamic hip screw (DHS) in the treatment of FNF.

**Methods:**

Fifty cases of FNF from July 2018 to February 2020 were retrospectively analyzed. According to different treatment methods, they were divided into DCLS group (26 cases) and DHS group (24 cases). Baseline data, intraoperative and postoperative clinical data, reoperation rate, and Harris score were collected to evaluate the clinical efficacy.

**Results:**

All patients were followed up for 24 months. All “fractures” were caused by fall. The baseline data of the two groups were comparable (*P* > 0.05). There weren’t significant differences in the length of hospital stay and mobility after two years postoperatively between the two groups (*P* > 0.05). The operation time, blood loss, incision length, fluoroscopy times and the degree of femoral neck shortening after two years postoperatively in the DCLS group were significantly less than those in the DHS group (all *P* < 0.05). Harris score after two years postoperatively in the DCLS group was significantly higher than that in the DHS group (*P* < 0.05). Although the reoperation rate in the DHS group was slightly higher than that in the DCLS group, it wasn’t statistical significance (*P* > 0.05).

**Conclusions:**

Compared with DHS, DCLS in the treatment of FNF had less surgical trauma, shorter incision length, shorter operation time, lower radiation dose and higher Harris scores. Although the reoperation rate in the DHS group was slightly higher than that in the DCLS group, it wasn’t statistical difference. Further research is needed.

## Background

Femoral neck fractures (FNF) accounts for about 57% of hip fractures, and has become one of the major health problems with increasing incidence and economic burden year by year [1, 2]. Dysfunction of hip and decreased quality of life increase patients’ mortality [3]. Most of these injuries require surgery treatment to prevent bedridden complications. For young patients, usually less than 60 years of age, joint replacement is usually not used because the implant life span usually does not last more than 20 years and multiple surgical complications will appear, such as infection, dislocation, and prosthesis loosening [4]. In addition, hip replacement is invasive, massively bleeding, and expensive, and often requires revision surgery after several years [5]. Physical status of these patients for hip replacement surgery has high requirements. The activities after hip replacement surgery are usually limited. So hip function after hip replacement surgery is often inferior to hip function before surgery. For young FNF patients, reduction and internal fixation is the preferred treatment, with the advantage of preserving autogenous femoral head [6].

The optimal fixation strategy for FNF is still controversial [7]. At present, no implant is superior to other implants in treating FNF [8]. The internal fixation methods for FNF include cancellous bone screws, dynamic hip screw (DHS), head nail, proximal femoral locking plate, etc. However, the postoperative failure rate of these fractures is very high, ranging from 20 to 80% [9]. Dynamic compression locking system (DCLS) is a new internal fixation system for FNF, which can apply axial, parallel, and uniform compression of fracture ends during surgery, be capable of sliding pressure after surgery, as well as form a stable frame structure [10]. DCLS has good initial and continuous stability. The DCLS is a potential choice for treatment of FNF with obvious advantages over three cannulated screws in clinical efficacy and biomechanics [11].

There is still a lack of consensus on which implant can better maintain the stability of fracture ends, promote fracture healing, and reduce postoperative femoral head necrosis and internal fixation failure [7]. Therefore, the purpose of this study was to compare the clinical efficacy of DCLS with DHS in the treatment of FNF in our hospital from July 2018 to February 2020, and to evaluate the strengths and weaknesses of the two implants.

## Methods

### Cases selection

Inclusion criteria were as follows: 1 A clear history of trauma; 2 A Garden II-III fracture of FNF; 3 Two years of follow-up after surgery; 4 Unilateral fractures; 5 The informed consents of the surgery and implant signed by these patients; 6 The applied implants of DCLS or DHS. 7 Closed reduction and internal fixation for the fracture treatment. Exclusion criteria were as follows: 1 A history of hip trauma at the same hip in the past; 2 Pathological fractures other than osteoporosis; 3 Senile dementia and other diseases with poor compliance and uncooperative treatment; 4 Concomitant fractures in other parts; 5 Concomitant acetabular fracture; 6 Serious medical disease interferes with clinical efficacy, such as paralysis or hemiplegia after cerebral hemorrhage or cerebral infarction, hepatic failure, renal failure, and serious cardiopulmonary dysfunction.

### General information

**Fig. 1:**
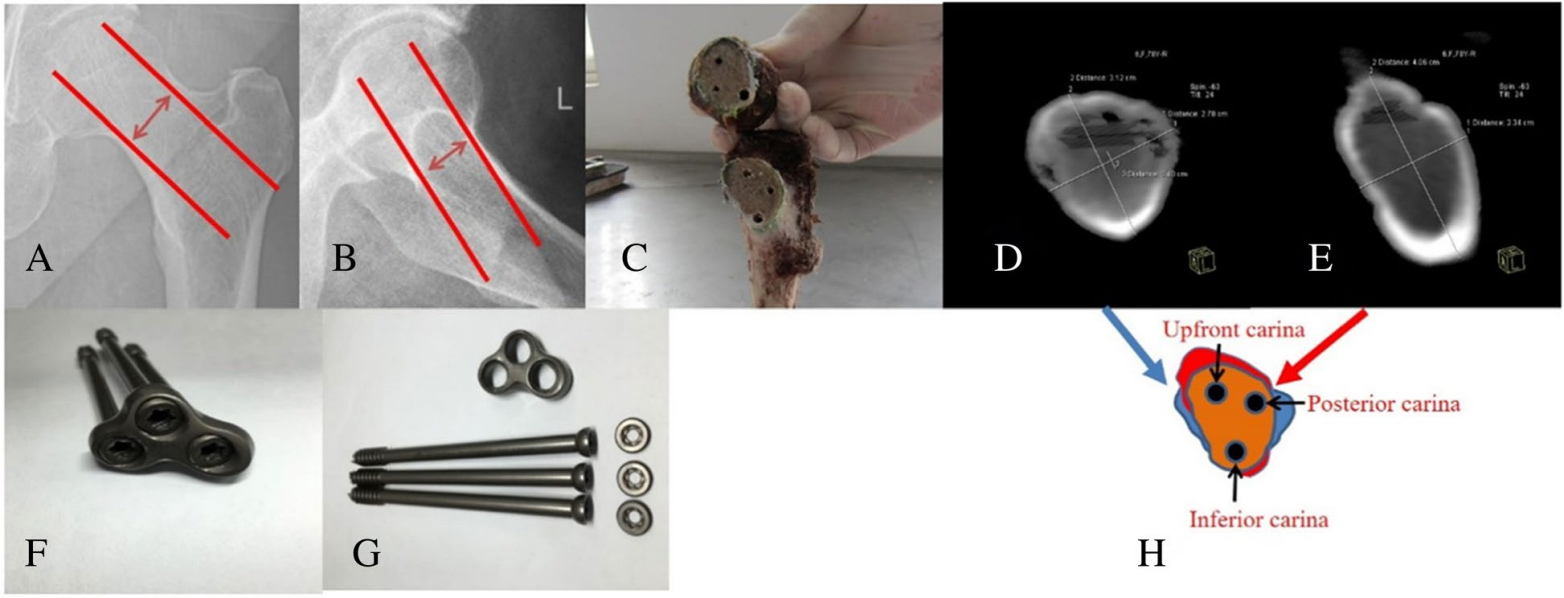
The triangular carina structure of the femoral neck and the composition of the DCLS system. A: The shortest distance between the upper and lower diameter of the femoral neck; B: The shortest distance between the anterior and posterior diameter of the femoral neck; C: The structure of the triangular carina and the position of the screws in the anatomical specimen (cited from our previous paper [10]); D: CT scan specimen showing cross-section of the minimum upper and lower diameter of the femoral neck; E: CT scan specimen showing the cross-section of the shortest distance of the anteroposterior diameter of the femoral neck; F and G: Components of the DCLS system (cited from our previous paper [10]); H: D + E cross-sections overlap to form a shared cross section of the femoral neck (saffron yellow area), triangular carina structure and screws placement (black circle)

The cases of FNF in our hospital from July 2018 to February 2020 were retrospectively collected. A total of 50 cases of FNF treated by DCLS (Suzhou Kangli Orthopaedic Medical Instrument Co. LTD, Jiangsu, China) (Fig. 1) and DHS (Shandong Weigao Orthopaedic Material Co. LTD, Shandong, China) fixation in our hospital were included. All fractures were caused by fall. According to the different treatment methods, the patients were divided into DCLS group (26 cases) and DHS group (24 cases). There weren’t statistical differences in preoperative clinical data (Table 1).

**Table 1:** Comparison of baseline data between the two groups

Data	DCLS group	DHS group	t/χ^2^	*P*
The number of cases	26	24	-	-
Age (years)	33-84(69.3±11.2)	32-89(68.5±11.9)	1.758	0.079
Gender			0.206	0.650
Male (cases)	8	6		
Female (cases)	18	18		
Time from injury to surgery (days)	0-5(2.7±1.4)	0-5(2.6±1.4)		
Garden classification (cases)				
II	6	6	0.017	0.986
III	20	18	0.025	0.874
Preoperative mobility(cases)			-	-
Walking without any aids	26	24		
Walking with crutches	0	0		
Walking with a walker	0	0		
Walking with a wheelchair	0	0		

### Surgical methods

All surgeries were performed by the same team of surgeons. These surgeons perform orthopedic surgery for years. We have completed about 120 cases of surgical treatment for femoral neck fracture every year, including about 30 cases of DCLS treatment, 30 cases of DHS treatment and 60 cases of hip replacement. The DCLS and DHS surgeries were performed under epidural anesthesia. These patients were supine on a traction bed. Closed reduction was performed under C-arm X-ray fluoroscopy. The criteria for satisfactory reduction were Garden index 160° to 175°, fracture displacement < 2 mm, and lateral angulation deformity < 10°. Surgical procedures in the DCLS group were based on previous studies [10, 11], and the surgical principles were shown in Fig. 1. DHS group was treated with DHS. The lateral side of the upper segment of the femur was chose as the approach to make a 6–10 cm surgical incision. The subcutaneous tissue and fascia were cut layer by layer to expose the trochanter and the upper segment of the femur. A 135° guide was selected to drill in the guide needle, which was directed towards the vertex of the femoral head. Intraoperative fluoroscopy ensured that the guide needle was located in the lower 1/3 of the femoral neck in the anterior and posterior fluoroscopy, and the middle of the femoral neck in the lateral fluoroscopy, with tip apex distance value < 25 mm. After the guide needle was in a good position, surgeon measured its length and screwed in the DHS main nail. After installing the lateral plate, surgeon installed corresponding screws in the lateral plate. Incision suture and drainage were routinely performed in both groups. Infection prevention was performed on all patients.

### Postoperative management

Ankle flexion and extension exercises were usually performed on the first day postoperatively. Patients at high risk of thrombosis were selectively treated with low-molecular weight heparin once daily for anticoagulant therapy until discharge. After discharge, these patients were instructed to take rivaroxaban orally for at least 1 month. After the third day postoperatively these patients began to do active ankle, hip and knee exercise. After 1–2 weeks postoperatively these patients were selectively started to walk with the aid of walking aids without weight-bearing. About 1 month after the surgery, these patients gradually started to walk with partial weight-bearing assisted by walking aids according to the patient’s condition. These patients gradually transitioned to full weight-bearing walking after 3 months postoperatively. These patients underwent X-ray review within 3 days after the operation. After discharge, these patients were instructed to make a routine out-patient re-visit at 1st, 2nd, 3rd and 6th month after the operation and follow up every 6 months thereafter until the fracture union completely.

### Observation index and evaluation criteria of curative effect

The hospital stay, operation time, intraoperative blood loss, incision length, intraoperative fluoroscopy times, femoral neck shortening length, hip joint Harris score, mobility and reoperation rate in 2 years postoperatively were collected and compared between the two groups. The length of femoral neck shortening was the difference between the affected side and the normal side measured on the anteroposterior radiograph of the pelvis. The measured value of femoral neck shortening was the vertical direction, that is, the difference between the normal side and the affected side from the center of femoral head to the greater trochanter. Ficat method was used to classify avascular necrosis of femoral head [12]. The hip function was evaluated according to Harris hip joint score [13]. Nonunion was judged by Dhar criteria [14]. Mobility was assessed according to a 4-level walking scale, namely walking without any aids, walking with crutches, walking with a walker, and walking with a wheelchair [15].

### Statistical analysis

SPSS 19.0 software (IBM Corporation, New York, USA) was used for statistical analysis. Count data were expressed in absolute logarithm and analyzed by χ^2^ test. Measurement data were expressed as mean ± standard deviation. Levene test was used to test homogeneity of variance of data. Independent sample T test was used for comparison between the two groups. *P* < 0.05 was considered statistically different.

## Results

All patients were followed up for 2 years (Table 2). There wasn’t significant difference in hospitalization time between the two groups (*P* > 0.05). The operation time, blood loss, incision length, times of fluoroscopy and the degree of femoral neck shortening in the DCLS group were significantly less than those in the DHS group (all *P* < 0.05). The Harris score of DCLS group was significantly higher than that of DHS group (*P* < 0.05). Femoral head necrosis occurred in 1 case and nonunion occurred in 1 case in the DCLS group, and all of them were re-operated later. In the DHS group, 3 cases of femoral head necrosis and 1 case of nonunion occurred, and all of them were re-operated later. Although the reoperation rate in the DHS group was slightly higher than that in the DCLS group, it wasn’t statistical significance (*P* > 0.05). For patients with osteonecrosis of the femoral head and nonunion, they would initially experience hip discomfort. Especially when the lower limb was weight-bearing, the hip discomfort symptoms would be aggravated, often accompanied by limited walking activity. Later these patients needed to use a walking aid for walking activities. Patients with osteonecrosis of the femoral head and nonunion had similar early symptoms and were indistinguishable by symptoms alone. But these patients can be confirmed by further CT and MRI examination of the hip. In the DCLS group, 1 case of postoperative mobility decreased by 1 grade and 1 case decreased by 3 grades, and the rest were the same as the preoperative mobility (Tables 1 and 2). However, 4 patients of the postoperative mobility in the DHS group were one grade lower than that of the preoperative, and the rest were the same as the preoperative mobility (Tables 1 and 2). There wasn’t significant difference in the postoperative mobility between the two groups (*P* > 0.05).

**Table 2:** Comparison of clinical data between the two groups

Data	DCLS group	DHS group	t/χ^2^	*P*
Length of stay (days)	15.3±4.2	15.0±4.2	1.034	0.301
Operation time (min)	59.7±9.2	78.3±10.1	-55.477	0.000
Blood loss (ml)	51.1±7.4	66.4±18.0	-29.906	0.000
Incision Length (cm)	4.1±0.48	7.3±1.07	-33.921	0.000
Fluoroscopy (times)	18.5±1.6	22.5±1.6	-38.531	0.000
Femoral neck shortening length (mm)	6.9±1.4	8.6±2.0	-9.466	0.000
Harris score	92.4±6.0	89.7±9.1	11.740	0.000
reoperation rate (cases)	2(7.7%)	4(16.7%)	0.952	0.329
Necrosis of femoral head	1(3.8%)	3(12.5%)		
Nonunion	1(3.8%)	1(4.2%)		
Postoperative mobility(cases)			3.089	0.213
Walking without any aids	24	20		
Walking with crutches	1	4		
Walking with a walker	0	0		
Walking with a wheelchair	1	0		

## Discussion

No matter which treatment method is chosen, FNF has a significant impact on the quality of life of these patients, and brings a greater economic burden to society and families. Compared with hip replacement, internal fixation has become the main treatment method for the undisplaced FNF due to its advantages of less trauma, shorter operation time, less bleeding and lower early mortality. The choice of surgical method should consider patient-related factors such as mobility, life expectancy, comorbidities and other fracture related factors such as fracture location, direction, and comminution [16]. The prognosis of FNF is uncertain. Bone nonunion and necrosis of femoral head are recognized as serious complications after internal fixation for FNF, which often require reoperation. The type of fracture and improper treatment are considered to be the main factors leading to nonunion and necrosis of femoral head [17]. However, there is still a lack of consensus on which internal fixation method can better maintain the stability of fracture ends, promote fracture healing, and reduce postoperative femoral head necrosis and internal fixation failure.

Three cannulated screws can be used for compression fixation of the fracture, but they do not lock each other to form a frame structure. The resistance of the three cannulated screws to vertical shear force and rotation force is relatively weak, which could lead to the loosening and displacement of the fracture ends, thereby increasing the risk of femoral head necrosis, nonunion and malunion [18–20]. The distribution pattern of cannulated screws was greatly affected by the subjective effect of the surgeons, so the clinical efficacy of cannulated screws in the treatment of FNF was significantly different between related studies. Previous studies have found that the DCLS treatment of FNF is superior to the three cannulated screws, with the advantages of small surgical trauma, good stability, early healing time, high fracture healing rate, early postoperative functional rehabilitation, low complication of fracture healing, and good recovery of hip function [11].

DHS fixation has the dual function of dynamic and static compression, so the fracture ends can contact closely. DHS can withstand twice the compressive stress of cancellous bone screws and have a higher fixation success rate. The lateral steel plate provides good angulation stability. The sliding mechanism of lag screws transforms the shear force into compressive stress, which is beneficial to fracture healing. However, it has been pointed out that its large trauma, long force arm, stress concentration and eccentric fixation may lead to fracture of locking plate and screw, re-fracture of femur, femoral head cutting and varus of hip [21]. Poor rotational stability, especially when the hip screw is screwing in the femoral head, is easy to cause poor rotational alignment between the femoral head and neck [22]. DHS requires greater soft tissue exposure and hip screw placement causes greater damage to the cancellous bone of the femoral head and neck, which disrupts the blood supply of the femoral head and neck and affects the healing of FNF.

DCLS is a new method of FNF fixation, which is in the initial clinical application stage. The main features are as follows. 1 The positions of the three parallel cannulated screws are distributed on the triangular carina of the section of the axial screw placement of the femoral neck. These screws are close to the bone cortex at the high bone density of the femoral neck, which conforms to the principle of “cortical support”. Therefore, these screws have good biomechanical stability with the characteristics of maximum screw dispersion and holding force [10]. 2 When the system inserts three cannulated screws, the three cannulated screws can apply axial and uniform pressure to the fractured ends through the lateral plate. 3 The system forms a triangular frame structure to resist shear and torsional forces. 4 The shortage of thread in the middle of these screws can generate dynamic and uniform compression capability of the fracture ends after operation to promote the healing of the fracture. Therefore, the functions of the DCLS system is the integration of three cannulated screws and DHS. The DCLS is further optimized and improved from three cannulated screws and DHS.

This study found that the operation time, blood loss, incision length, number of fluoroscopy, and shortening of the femoral neck in the DCLS group were significantly less than those in the DHS group, which indicates that the DCLS group had simpler intraoperative procedures, less trauma, and better control of femoral neck shortening than those in the DHS group. The DCLS is equipped with an intraoperative guide. After the first guide needle is placed in the femoral neck in a good position, the remaining guide needles can be operated with the guide, which simplifies the surgical process and improves the accuracy of screw placement. At the same time, the trauma and the number of intraoperative fluoroscopy are reduced, so the operation time and intraoperative radiation exposure of patients are reduced. Previous studies also found that femoral neck shortening was a common complication of FNF in the DHS group [23–25]. DCLS is locked into a triangular frame structure, which has good postoperative stability and can axially and evenly compress the fracture ends to promote fracture healing. On the other hand, the degree of shortening of the femoral neck is controlled to some degree so that it will not be excessively shortened.

In this study, 1 patient (3.8%) in the DCLS group suffered femoral head necrosis and 1 patient (3.8%) suffered nonunion. In the DHS group, 3 cases (12.5%) suffered femoral head necrosis and 1 case (4.2%) suffered nonunion. Although the complications in the DHS group were slightly higher than those in the DCLS group, it wasn’t statistical difference (*P* > 0.05). The Harris score in the DCLS group was significantly higher than that in the DHS group, but it hasn’t significant change in postoperative mobility between the two groups. A previous study found that about 11.3% of the cases of FNF in the DHS group suffered femoral head necrosis, and 9.4% of the cases suffered nonunion [26]. The rate of osteonecrosis of the femoral head in our study was similar to that of previous studies, but the nonunion rate was significantly lower than that in previous studies, which may be related to the study population, fracture type, reduction quality, and operators. Several studies have also found that DHS combined with anti-rotation screws for displaced FNF can prevent rotational displacement of the femoral head during hip screw placement, which could increase biomechanical stability of better mechanical support, shorter operative time, less radiation exposure, and higher hip Harris score [22, 23]. DHS technique and cannulated cancellous screws technique are the two main fixation techniques for the treatment of FNF. The Meta-analysis study found that the nonunion rate of the cannulated cancellous screws group was significantly higher than that of the DHS group, but it hasn’t significant difference in the incidence of femoral head necrosis between the two groups. For vertically oriented FNF, the DHS technique is more favorable than the cannulated cancellous screws technique, with a lower risk of nonunion [27]. The DCLS group had a higher Harris score, a lower incidence of femoral head necrosis than those in the DHS group, which may be related to the excellent characteristics such as the better stability of the “cortical support”, triangular frame structure, intraoperative uniform compression and postoperative uniformly dynamic pressure.

### Limitations

In this study, the rate of re-operation in the DCLS group was lower than that in the DHS group, but it hasn’t statistical difference, which might be limited and related with the small number of cases in these groups. So further large sample research is needed. The retrospective analysis is prone to data bias. Moreover, this study was a preliminary single-center study of this system. In order to further verify the clinical efficacy of DCLS, further multi-center randomized, controlled, and double-blind clinical trials are required.

## Conclusions

Compared with DHS, DCLS in the treatment of FNF has less surgical trauma, shorter incision, shorter operation time, lower radiation dose and higher Harris scores. Although the reoperation rate in the DHS group was slightly higher than that in the DCLS group, it wasn’t statistical difference. Further research is needed.

## Data Availability

The datasets used and/or analysed during the current study are available from the corresponding author on reasonable request.

## Abbreviations

FNF: Femoral neck fractures
DCLS: Dynamic compression locking system
DHS: Dynamic hip screw
